# General Condition for Polymer Cononsolvency in Binary Mixed Solvents

**DOI:** 10.1021/acs.macromol.4c00246

**Published:** 2024-08-20

**Authors:** Xiangyu Zhang, Jing Zong, Dong Meng

**Affiliations:** † Biomaterials Division, Department of Molecular Pathobiology, New York University, New York, New York 10010, United States; ‡ Department of Chemical and Biomolecular Engineering, John Hopkins University, Baltimore, Maryland 21218, United States; § Department of Chemical Engineering, Mississippi State University, Mississippi State, Mississippi 39762, United States

## Abstract

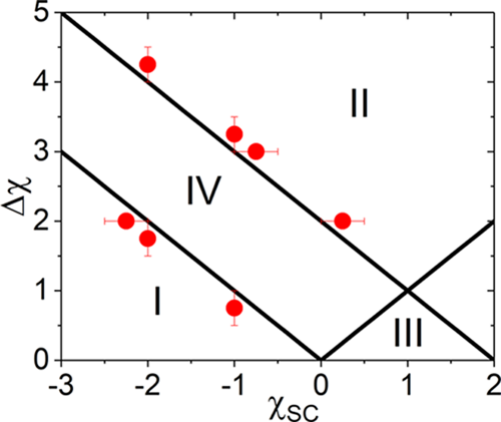

Starting from a generic model based on the thermodynamics of mixing and abstracted from the chemistry and microscopic details of solution components, three consistent and complementary computational approaches are deployed to investigate the general condition for polymer cononsolvency in binary mixed solvents at the *zeroth* order. The study reveals χ_PS_ – χ_PC_ + χ_SC_ as the underlying universal parameter that regulates cononsolvency, where χ_αβ_ is the immiscibility parameter between the α- and β-component. Two disparate cononsolvency regimes are identified for χ_PS_ – χ_PC_ + χ_SC_ < 0 and χ_PS_ – χ_PC_ + χ_SC_ > 2, respectively, based on the behavior of the second osmotic virial coefficient at varying solvent mixture composition *x*_C_. The predicted condition is verified using self-consistent field calculations by directly examining the polymer conformational transition as a function of *x*_C_. It is further shown that in the regime χ_PS_ – χ_PC_ + χ_SC_ < 0, the reentrant polymer conformation transition is driven by maximizing the solvent-cosolvent contact, but in the regime χ_PS_ – χ_PC_ + χ_SC_ > 2, it is driven by promoting polymer and cosolvent contact. In-between the two regimes when neither effect is dominant, a monotonic response of polymer conformation to *x*_C_ is observed. Effects of the mean-field approximation on the predicted condition are evaluated by comparing the mean-field calculations with computer simulations. It shows that the fluctuation effects lead to a higher threshold value of χ_PS_ – χ_PC_ + χ_SC_ in the second regime, where local enrichment of cosolvent in polymer proximity plays a critical role.

## 1 Introduction

Solution processing is a fundamental process in polymer science in which solvent mixtures are often in use. If two good solvents become poor for a polymer when mixed, then the solvent mixture is called a cononsolvent pair for the polymer. The cononsolvency effect had been observed for many types of polymers in a variety of solvent mixtures, characterized by either reentrant coil–globule–coil (C-G-C) conformational transition^1−3^ and/or reentrant mixing-demixing-mixing phase transition with respect to the solvent mixture composition.^4,5^ Computer simulations incorporating atomistic details had revealed a multitude of system-dependent molecular mechanisms that can give rise to the phenomenon, ranging from competitive/cooperative/frustrated hydrogen bonding of polymer molecules with polymer/solvent/cosolvent molecules^4,6−9^ to formation of solvent/cosolvent complexation cluster,^10−14^ polymer-mediated S–C interactions, and alteration of the solvent structuring by the presence of cosolvent,^15,16^ etc. Recent research interests have been directed toward seeking a generic understanding on the driving forces and mechanism of polymer cononsovlency.^17−20^ Early studies predicted polymer cononsolvency when solvents and cosolvents are strongly associative and have negative excess free energy of mixing (e.g., water/DMSO mixture).^2,21−23^ The explanation however struggles with polymer cononsolvency observed in water–alcohol mixtures, which are known to have positive excess free energy of mixing.^24^ Recent studies by Mukherji’s et al. claim that preferential adsorption of cosolvent by polymer is responsible for cononsolvency of PNIPAM in water–methanol mixtures.^25^ The authors demonstrate the generic nature of the preferential-adsorption effect by showing that atomistic simulations of PNIPAM in water–methanol mixtures are in excellent agreements with the coarse-grained simulations that are deprived of all chemistry details.^26^ A generic adsorption model is further proposed to show that competition of two adsorption modes of cosolvents by polymer toward minimizing the adsorption free energy leads to a reentrant polymer conformation transition with respect to solvent mixture composition. The concept of preferential adsorption has since been used for the developments of several theoretical models by introducing ad hoc “bridging” enthalpy between polymer and cosolvent.^27−29^ Recently, understanding of the two types of polymer cononsolvency under one unified generic formulation had been discussed by us.^30^ Early investigations have reinforced the plausibility that polymer cononsolvency is governed by general principles that are chemistry nonspecific, although mechanisms at the molecular level may vary.

Despite the progress, the general condition for polymer cononsolvency that applies to a broad variety of systems has not yet been clearly presented. This leads to doubts and confusion about the validity of the generic considerations. For example, numerous experiment studies had reported that preferential adsorption of cosolvents by polymer does not necessarily give rise to polymer cononsolvency.^31,32^ Using coarse-grained simulations, a recent study by Bharadwaj et al. shows that polymer C-G-C conformation transition can occur under a myriad of different settings, including strong solvent-cosolvent energetic interactions, solvent-cosolvent size difference, and strong solvent–solvent energetic interactions.^33^ They proceed to conclude that neither solvent-cosolvent energetic attractions nor preferential adsorption of cosolvent by polymer warrants or are prerequisites for polymer cononsolvency. These seemingly contradictory findings highlight two outstanding needs toward an improved understanding of the phenomena based on generic considerations. First, current generic understandings seem to suggest separate cononsolvency conditions depending on the driving force (e.g., “solvent-cosolvent attractions” vs “preferential adsorption”). There needs to be a unified formulation of the condition that naturally governs polymer cononsolvency of seemingly distinct origins. In order to do this, the relevant underlying parameter that regulates polymer.cononsolvency needs to be identified. For it to apply to a broad variety of systems, the parameter would likely be of a thermodynamic nature. Microscopic interactions (of either enthalpic or entropic origins) are certainly not suitable choices. On the other hand, “preferential adsorption” as a thermodynamic phenomenon refers to the corresponding excess cosolvent density in the solvation shell. However, it is premature to expect the onset of preferential adsorption to commence the reentrant polymer conformation transition.

In view of the needs, in this study, we make the attempt to determine the underlying governing parameter and the corresponding condition for polymer cononsolvency in binary mixed solvents. A generic formulation based on the thermodynamics of mixing was used to keep the results independent of the microscopic details. Three separate but complementary approaches will be combined to achieve the task. An analytical theory offers a clear mathematical path to derive the general condition and identify the relevant parameters. Using the same model, field theoretical calculations provide direct access to polymer conformation transitions for the verification of the analytical predictions. Effects of the mean-field approximations on the theoretical predictions will be assessed and examined in computer simulations. The manuscript is organized as the following. Section 2 gives an account of the development of the model and methods employed in this work. In Section 3, the conditions for polymer cononsolvency are first predicted analytically based on the behavior of the second osmotic virial coefficient, followed by a study of polymer conformation transitions under the corresponding conditions to verify and rationalize the predictions. We then compare the results with computer simulations to estimate the effects of mean-field approximations on the predicted conditions. In Section 4, we conclude with a discussion of the implications of the current study for rationalizing available experiments and simulation results and then touch on the possible improvements that can be made to the current approach.

## 2 Model and Methods

We aim at a generic model that contains the essential ingredients for producing the gross character of polymer cononsolvency. Based on the model, three separate but complementary approaches will be developed and combined to derive and verify the general conditions for polymer cononsolvency. Toward this, we develop the model from a thermodynamic perspective that involves less hypothesis concerning the structure and laws of interactions between molecular species. The starting point is a generalized, however, exact expression of the Helmholtz free energy per unit volume of a ternary system containing polymer (P), solvent (S), and cosolvent (C).
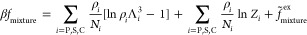
1

The first sum accounts for the contribution from the translational entropy of the three components, with ρ_*i*_ being the number density of the *i*-type monomer in the mixture, *N*_*i*_ is the degree of polymerization (*N*_S_ = *N*_C_ = 1) and Λ_*i*_ is the thermal de Broglie wavelength for the degrees of freedom of center of mass of an *i*-type molecule. The second term arises from the conformation entropy due to the internal degree of freedom of molecules, with *Z*_*i*_ being the reference ideal-state conformation partition function of an *i*-type molecule. The last term *f̃*_mixture_^ex^ is the excess free energy per unit volume due to all of the interaction effects in the mixture. By assuming no volume change upon mixing (i.e., *V* = ∑_*i*_*V*_*i*_^(0)^ with *V*_*i*_^(0)^ being the volume of the *i*-component in its pure state), a formally exact expansion of the excess free energy of mixing per unit volume can be obtained (see Section 5 for details) as
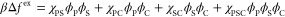
2where χ_*ij*_ and χ_*ijk*_ are expansion coefficients of free-energy nature that are in general ϕ-dependent.

The models for this study are based on eq 2. We restrict our considerations to the zero-order case; that is, the χ-parameters are assumed to be ϕ-independent. For simplicity, we also neglect the nonaddititive effects χ_PSC_. Considering a homogeneous system where ϕ_*i*_ is location-independent, inclusion of change in translational entropy of molecular center of mass into eq 2 (change in polymer conformational entropy due to mixing is contained in χ-parameters) yields the free energy of mixing
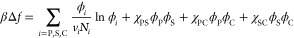
3which has the form of the Flory–Huggins (F–H) theory. Considering an inhomogeneous system with ϕ_*i*_(**r**) being the volume fraction of component *i* at location **r**, the effective Hamiltonian of a particle-based model can be constructed based on eq 2. Specifically, the *local* excess free energy of mixing in volume element d**r** at location **r** can be written as d**r**ρ_0_(**r**)Δ*f*^ex^, where ρ_0_(**r**) is the total packing fraction of the *effective* particles (referred to hereafter as segments) at location **r**. Dropping the χ_*ijk*_-term in eq 2 for consistence, the effective Hamiltonian becomes
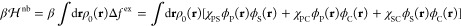
4Upon introducing the segmental microscopic densities ρ̂_*i*,*c*_(**r**′) ≡ ∑_*k*_δ(**r**′ – **r**_*i*,*k*_) where **r**_*i*,*k*_ is the center location of the *k*-th segment of type *i*, ρ_0_(**r**) and ϕ_*i*_(**r**) in eq 4 can be written as ρ_0_(**r**) = ∑_*i*=P,S,C_ρ̂_*i*_(**r**) and , respectively, where ρ̂_*i*_(**r**) = ∫d**r**′*S*_*i*_(**r** – **r**′)ρ̂_*i*,*c*_(**r**′) and *S*_*i*_(**r** – **r**′) is the shape function of the *i*-type segment that satisfies ∫d**r***S*_*i*_(**r** – **r**′) = *v*_*i*_. For a strictly incompressible system, ρ_0_(**r**) = ρ_0_ and eq 4 becomes
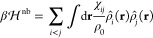
5

By defining *u*_*ij*_(**r**′ – **r**″) ≡ ∫d**r***S*_*i*_(**r** – **r**′_*i*_)*S*_*j*_(**r** – **r**″_*j*_), a pairwise form is arrived
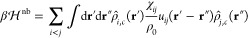
6in which  acts as the effective interaction between segment *i* and *j* (*i* ≠ *j*) due to immiscibility. In the particle-based model, the incompressibility condition  is approximated by including into  an excluded volume potential  with κ ≫ χ_*ij*_ between all pair centers. The final form of  then becomes
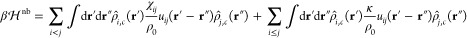
7for convenience, we explicitly specify the function form for *u*_*ij*_(**r** – **r**′) while keeping *S*_*i*_(**r** – **r**′) implicit. For all *ij*-pair we set
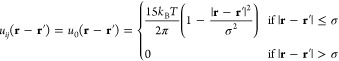
8The center locations of effective segments introduced in eq 5 are also used to construct the effective Hamiltonian arising from polymer chain connectivity, i.e.
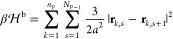
9where **r**_*k*,*s*_ is the center location of the *s*-th segment on the *k*-th polymer chain. Equations 7–9 define an effective particle-based model that is consistent with eq 2, and in which polymer conformational degree of freedom beyond the segment scale in an inhomogeneous environment can be explicitly accounted for. High κ values (κ = 16 throughout this study) are employed in implementations of the particle-based models to ensure near incompressibility so that the χ-parameters are comparable to that in the F–H model. For simplicity, we also assume the same segmental volume for all types of segments (*v*_*i*_ = *v*_0_, *i* = P, S, C) in the calculations. The F–H theory will be used to derive an analytical expression of the second osmotic virial coefficient from which the conditions for polymer cononsolvency are determined. Using the effective particle model, self-consistent field (SCF) calculations will be conducted to examine the polymer conformational transition as a function of solvent mixture composition to verify the predicted condition. Monte Carlo (MC) simulations will be used to assess the effects of mean-field approximation on the predictions with the inclusion of fluctuation effects, which prove to be important in dilute polymer solutions. Details of the implementations can be found in Section 5.

## 3 Results

### Second Osmotic Virial Coefficient 

3.1

**Figure 1:**
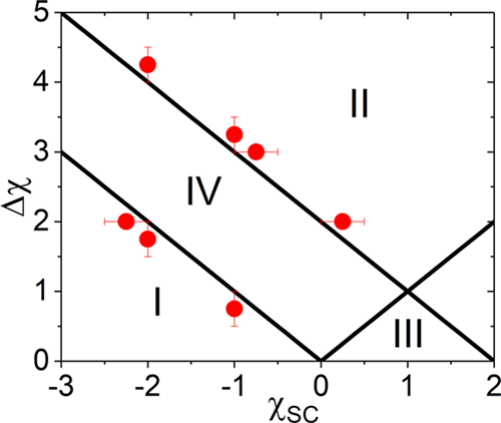
Respective regions in the χ_SC_ – Δχ plane in which , derived from the F–H theory, varies monotonically (IV), possesses minimum (I and II), or maximum (III) with respect to *x*_C_. Symbols are estimated boundary points separating the monotonic and reentrant polymer conformation transitions in the SCF calculations.

For a sufficiently dilute polymer/solvent/cosolvent solution (ϕ_P_ ≪ 1), the second osmotic virial coefficient can be derived as  (see Section 5 for derivation), where  corresponds to the second osmotic virial coefficient in binary polymer/solvent mixtures, and the term
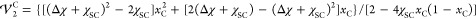
10accounts for the cosolvent effects with *x*_C_ ≡ ϕ_C_/(ϕ_C_ + ϕ_S_). The dependence of  on *x*_C_ derives solely from  which is controlled by two parameters: the solvent/cosolvent immiscibility parameter χ_SC_ and Δχ ≡ χ_PS_ – χ_PC_ that characterizes the immiscibility mismatch between polymer/cosolvent and polymer/solvent. With no loss of generality, we let Δχ ≥ 0, i.e., cosolvent is defined as the species that is more miscible with polymer. Equation 10 gives  and Δχ at *x*_C_ = 0 and 1, respectively. Solving the equation  yields two extrema for  at  and  subject to the constraints of *x*_C,1_^*^ ∈ [0, 1] and *x*_C,2_^*^ ∈ [0, 1]. Depending on the presence and nature of the extrema in , the χ_SC_ – Δχ plane can be divided into four regions as shown in Figure 1.  possesses a minimum (<0) in region I and II, a maximum in region III, but is monotonic in region IV. In the overlapping region between II and III,  shows both a minimum and a maximum. Since  manifests the strength of effective repulsions between polymer segments, an inverse response of  in regions I and II with increasing *x*_C_ implies a reentrant type polymer conformational transition. For this reason, we define regions I and II as the two cononsolvency regimes in this study, and their exact boundaries are determined as
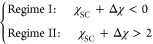
11respectively. Contrastingly,  exhibits an overshoot in response to increasing *x*_C_ in region III which can be accordingly defined as the cosolvency region.^22^ We defer the discussion of cosolvency to a future study and will focus on cononsolvency in this work. It is worth mentioning that the boundaries in Figure 1 depend on Δχ = χ_PS_ – χ_PC_ but not on χ_PS_ and χ_PC_ individually. At fixed Δχ, changing χ_PS_ only has the effect of shifting  by the *x*_C_-independent . In comparison, the monomer chemical potential μ_P_ can be derived from eq 3 as
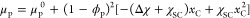
12where  is the monomer chemical potential in binary P/S mixtures, and the second term arises from the cosolvent effects. It can be shown that in Regime I (χ_SC_ + Δχ < 0), μ_P_ changes synchronously with , possessing a maximum with respect to *x*_C_ ∈ [0, 1] at  where  is at its minimum. In Regime II (χ_SC_ + Δχ > 2), however, μ_P_ decreases *monotonically* with respect to *x*_C_ ∈ [0, 1] whereas  possesses a minimum, indicating a decoupled behavior of μ_P_ and  in this region.

### Polymer Conformation 

3.2

**Figure 2:**
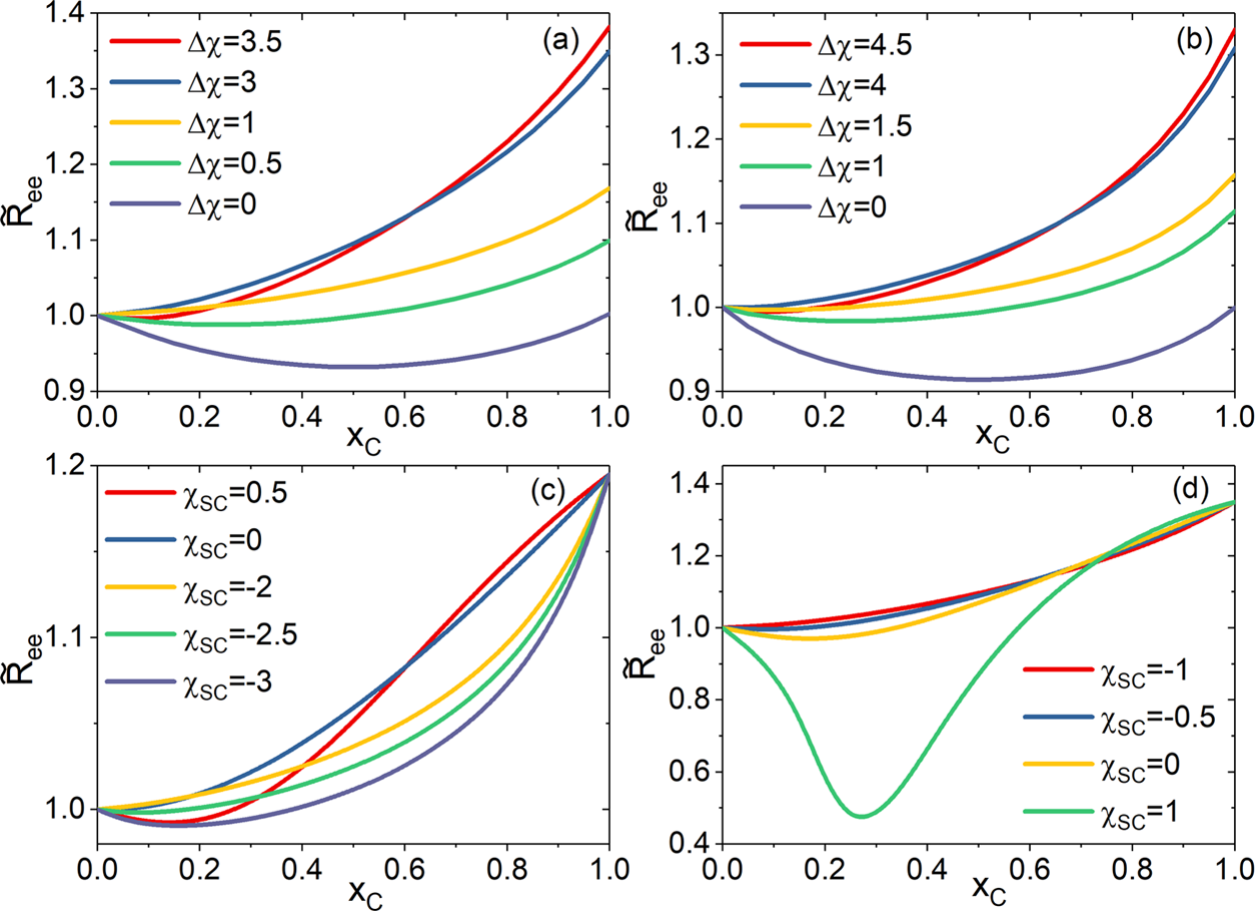
Reduced polymer end-to-end distance  obtained from the SCF calculations at: (a) χ_SC_ = −1, χ_PS_ = 0.5, Δχ = 0 → 3.5; (b) χ_SC_ = −2, χ_PS_ = 0, Δχ = 0 → 4.5; (c) Δχ = 2, χ_PS_ = 0, χ_SC_ = −3.0 → 0.5; and (d) Δχ = 3, χ_PS_ = 0.5, χ_SC_ = −1.0 → 1.0.

Different behaviors by  in response to *x*_C_, as described by Figure 1, imply distinct polymer conformational responses (monotonic vs reentrant) in the respective regions. This can be directly examined and verified in the SCF calculations at the mean-field level. Given the large parameter space, the SCF calculations are carried out with the chain length *N* = 80 for a selected set of parameters to determine the reduced polymer end-to-end distance  as the function of *x*_C_. Figure 2(a–d) shows  calculated at: (a) χ_SC_ = −1, χ_PS_ = 0.5, Δχ = 0 → 4.0; (b) χ_SC_ = −2, χ_PS_ = 0, Δχ = 0 → 5.0; (c) Δχ = 2, χ_PS_ = 0, χ_SC_ = −3.0 → 1.0; and (d) Δχ = 3, χ_PS_ = 0.5, χ_SC_ = −1.0 → 1.0. In Figure 2(a), at Δχ = 0,  shows a reentrant behavior symmetric about *x*_C_ = 0.5 where a minimum is observed. Increasing Δχ from zero gradually lessens the magnitude of the minimum in  until it turns into a monotonically increasing function of *x*_C_ at Δχ = 1. As Δχ further increases to 3.5, reentrant behavior of  re-emerges and intensifies toward greater Δχ. A similar trend is seen in Figure 2(b) as Δχ increases with χ_SC_ = −2 and χ_PS_ = 0. Figure 2(c,d) shows how the transition of  varies as χ_SC_ increases at fixed Δχ. With Δχ = 2 and χ_PS_ = 0,  changes from a reentrant behavior at χ_SC_ = −3 to a monotonic behavior when χ_SC_ = −2, and back to a reentrant behavior again at χ_SC_ = 0.5. With Δχ = 3 and χ_PS_ = 0.5,  stays monotonic until χ_SC_ = −1.0 when a reentrant behavior starts to appear and keeps intensifying with further increasing χ_SC_. The boundary values separating the monotonic and reentrant behaviors by  can be estimated from Figure 2(a–d) as (a) (χ_SC_ = −1, Δχ = 0.5 – 1) and (χ_SC_ = −1, Δχ = 3 – 3.5); (b) (χ_SC_ = −2, Δχ = 1.5 – 2.0) and (χ_SC_ = −2, Δχ = 4 – 4.5); (c) (Δχ = 2, χ_SC_ = −2.5 to −2) and (Δχ = 2, χ_SC_ = 0 – 0.5); and (d) (Δχ = 3, χ_SC_ = −1 to −0.5). The estimated boundaries, obtained with two different χ_*PS*_ values, are in agreement with the predictions by eq 11 (Figure 1), further attesting the relevance of Δχ + χ_SC_ in formulating the general conditions for cononsolvency.

**Figure 3:**
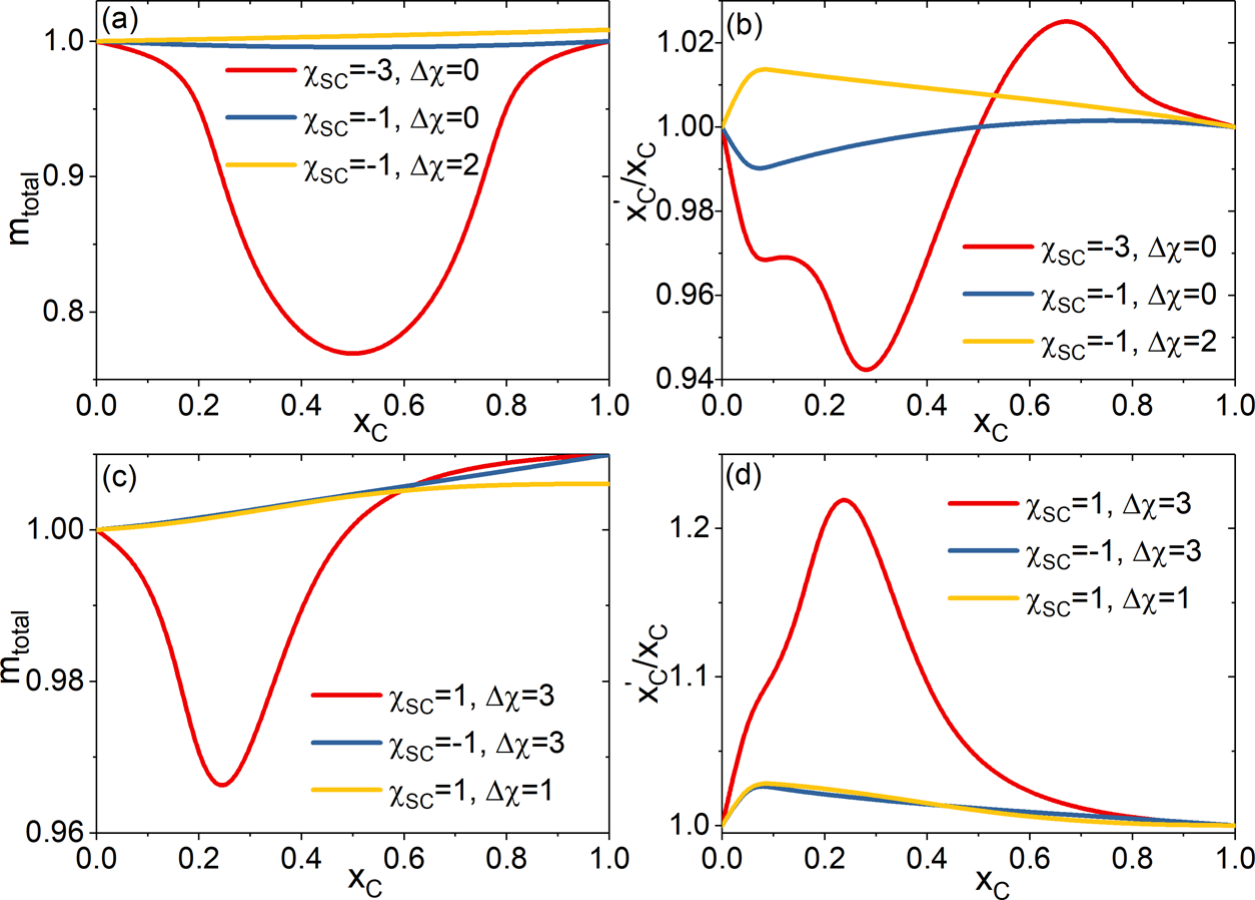
(a) and (c): Total polymer–solvents contact *m*_total_; (b) and (d): the local cosolvent enrichment *x*_C_^′^/*x*_C_ as the function of *x*_C_ at a set of Δχ and χ_SC_ values. See the text for details.

We now seek further insights into the role of Δχ and χ_SC_ in inducing reentrant polymer conformational transitions. Toward that, we examine the local solution structure in polymer proximity which can be characterized by the overall polymer-(co)solvent contact and the local cosolvent composition. The contact between the polymer and the α-solvent can be quantified as *m*_Pα_ ≡ ρ_0_*E*_Pα_/χ_Pα_ (α = S and C) with *E*_Pα_ being the polymer-α interaction energy. The total polymer-(co)solvent contact and the local solvent mixture composition can then be accordingly calculated as *m*_total_ = *m*_PC_ + *m*_PS_ and *x*_C_^′^ = *m*_PC_/*m*_total_, respectively. Figure 3(a,b) shows *m*_total_ and *x*_C_^′^/*x*_C_ as the function of *x*_C_ at a set of Δχ and χ_SC_ values, respectively. With Δχ = 0 and χ_SC_ = −3 (inside Regime I), Figure 3(a) shows that *m*_total_(*x*_C_) exhibits a reentrant behavior symmetric about *x*_C_ = 0.5 that tracks the transition of . On the other hand, the local cosolvent composition *x*_C_^′^ shown in Figure 3(b) indicates a depletion of the minor solvent component (i.e., *x*_C_^′^ < *x*_C_ when *x*_C_ < 0.5 and *x*_C_^′^ > *x*_C_ when *x*_C_ > 0.5). This depletion signals that solvent-cosolvent contact is being promoted in the solution by moving the minor solvent species away from polymer proximity and replacing them with the primary solvent.

As *x*_C_ → 0.5, this tactic becomes increasingly less effective, as there are not enough primary solvents to keep both the minor solvent and polymer in accompany. As the result, promoting S–C contact relies increasingly on polymer size contraction, which explains  approaching minimum at *x*_C_ = 0.5. Therefore, the polymer conformation transition in Regime I can be understood as being driven by system maximizing solvent-cosolvent contact. In consequence, greater χ_SC_ or Δχ values will act to reduce this driving force, leading to lesser to none depletion by the minor solvent specie as shown in Figure 3(b). This results in the eventual disappearance of reentrant transition by  as the system moves out of Regime I. As comparison, with Δχ = 3 and χ_SC_ = 1 (inside Regime II), Figure 3(d) shows that the local cosolvent composition *x*_C_^′^ exhibits a pronounced peak at *x*_C_ ∼ 0.28 corresponding to *a* > 20% increase from the bulk cosolvent composition. The peak compares well with a similar extent of contraction in  shown in Figure 2, but contrasting to only ∼3% change in *m*_total_ shown in Figure 3(c). This suggests that unlike in Regime I where the overall contact between polymer and (co)solvent is reduced in favor of solvent-cosolvent contact, polymer contraction in Region II is driven by promoting the polymer-cosolvent contact through partitioning of cosolvent molecules in favor of polymer proximity. The extent of cosolvent localization, characterized by *x*_C_^′^/*x*_C_, depends on the competitive interplay of entropy of mixing and favorable energetics. Strongly favorable energetics are required to overcome the entropic penalty to generate local cosolvent-enriched regions at low *x*_C_, which induces polymer conformational contraction. Effectively favorable energetics for polymer-cosolvent contact can derive from excess affinity of the cosolvent to the polymer and/or greater solvent-cosolvent immiscibility. This means that greater Δχ and χ_SC_ values will lead to more pronounced peak in *x*_C_^′^ (Figure 3(d)) and drive the system toward Regime II. The above analysis rationalizes the emergence of Δχ + χ_SC_ as the relevant parameter in the general condition predicted in eq 11. The two cononsolvency regimes are located on opposite ends of the Δχ + χ_SC_ scale, separated by a monotonic region as the driving force for polymer conformation transition changes from maximizing S/C contact to promoting P/C contact.

**Figure 4:**
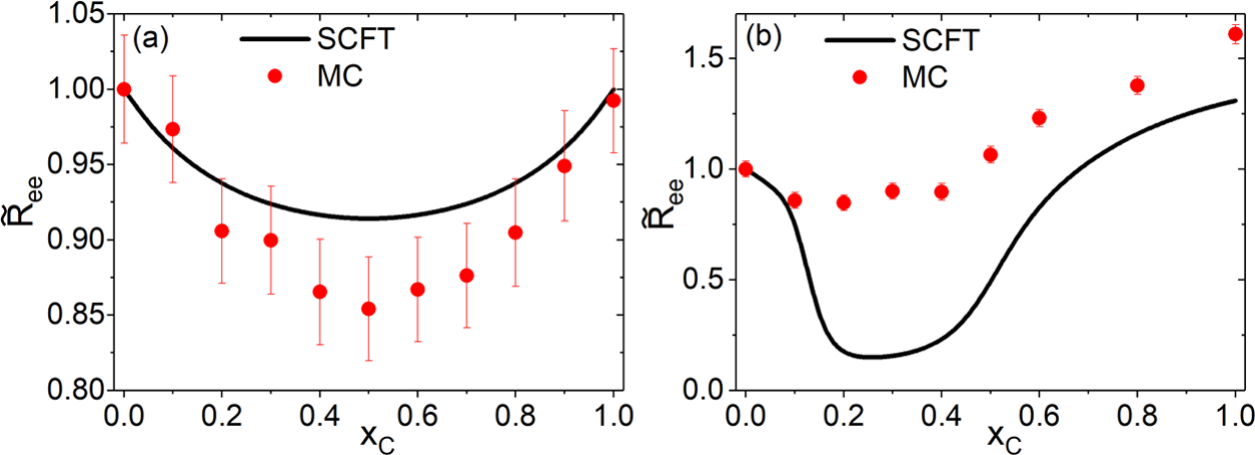
Comparison of  obtained from the SCF calculations and MC simulations at (a) Δχ = 0 and χ_SC_ = −2, and (b) Δχ = 4 and χ_SC_ = 1.

**Figure 5:**
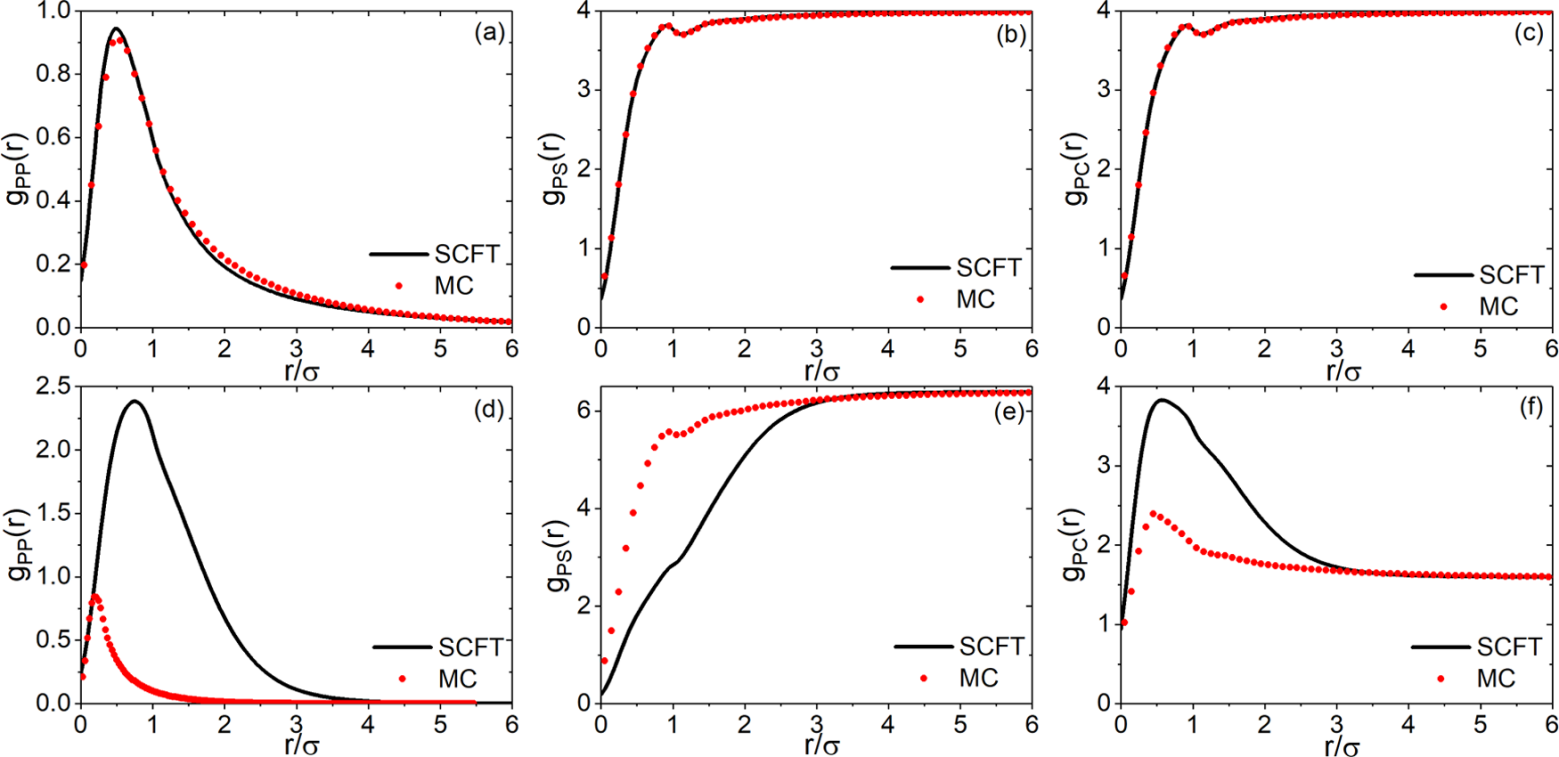
Comparison of radial distribution functions from the SCF calculations and MC simulations at (a–c) Δχ = 0 and χ_SC_ = −2 with *x*_C_ = 0.5, and (d–f) Δχ = 4 and χ_SC_ = 1 with *x*_C_ = 0.2.

The effect of the mean-field approximation (employed in the Flory–Huggins and SCF calculations) on the predicted general condition can be assessed by comparison to MC simulations. Using the same model, such comparisons will reveal unambiguously the effects of fluctuating local solvent mixture compositions on the reentrant polymer conformation transition. Figure 4(a,b) compares  obtained from the SCF calculations and MC simulations. At χ_SC_ = −2 and Δχ = 0 (χ_PS_ = 0, χ_PC_ = 0) (inside Regime I), the two results are in semiquantitative agreement, showing the reentrant behavior of  being symmetric about *x*_C_ = 0.5 and the extent of contraction being slightly underestimated by the SCF calculations. In contrast, at χ_SC_ = 1 and Δχ = 4 (χ_PS_ = 0, χ_PC_ = −4) (inside Regime II), Figure 4(b) shows that the SCF calculations substantially overestimate the extent of  contraction comparing to the MC simulations. Figure 5 compares the radial distribution functions obtained from the SCF calculations and MC simulations for the two cases in Figure 4(a–b) at *x*_C_ = 0.5 and 0.2 where  reaches their respective minimum. Again, good agreements are seen in all three pairs of radial distributions at χ_SC_ = −2 and Δχ = 0. But at Δχ = 4 and χ_SC_ = 1, a much higher degree of cosolvent enrichment around polymer is predicted by the SCF calculations. The results suggest that the influence of fluctuation effects on the reentrant polymer conformation transition is much stronger in Regime II than in Regime I. This is probably because, as discussed above, the reentrant transition inside Regime II relies on the local cosolvent enrichment in polymer proximity, which is more subject to the effects of composition fluctuations, whereas the reentrant transition inside Regime I is driven by promoting solvent-cosolvent contact, which maximizes at *x*_C_ = 0.5 and takes place dominantly in the bulk region far from the polymer. We therefore conclude that the mean-field approximation leads to an underestimate of the threshold value of χ_SC_ + Δχ for Regime II, but has much less effects on that for Regime I.

## 4 Summary and Discussion

Starting from a generic framework based on the thermodynamics of mixing and abstract from the chemistry details of solution components, a model is developed and studied using three complementary computational approaches to investigate the general condition for polymer cononsolvency in binary solvent systems. Our study shows Δχ + χ_SC_ as the underlying universal parameter that regulates cononsolvency. Two disparate cononsolvency regimes are identified for Δχ + χ_SC_ < 0 and Δχ + χ_SC_ > 2, respectively, based on the behavior of the second osmotic virial coefficient . These conditions are further verified in the self-consistent field calculations by directly examining the polymer conformation transitions. It is further shown that in the regime Δχ + χ_SC_ < 0, reentrant polymer conformational transition is driven by maximizing the solvent and cosolvent contact, but in the regime Δχ + χ_SC_ > 2, it is driven by promoting polymer and cosolvent contact. In-between, when neither effect is dominant, a monotonic response of polymer conformation to cosolvent composition is observed. Results from the mean-field calculations are in qualitative agreement with the Monte Carlo simulations. Effects of compositional fluctuations are found to play a more significant role in the Δχ + χ_SC_ > 2 regime, suppressing local enrichment of the cosolvent in polymer proximity.

Results of this study rationalize trends observed in several experiments and computer simulations. In particular, connections can be made with reference to the three microscopic cononsolvency models discussed in ref (33). In the model that features dominant solvent-cosolvent energetic attraction, the settings of the microscopic simulations corresponds to having χ_SC_ < 0 and Δχ ≡ χ_PS_ – χ_PC_ > 0 in the current formulation. Given enough solvent-cosolvent attraction strength (χ_SC_ being more negative), χ_SC_ + Δχ turns negative and places the system inside Regime I, where reentrant conformation transition is accompanied by depletion of the minority solvent specie (similar to what is shown in Figure 2(b)). Increasing monomer-cosolvent attraction strength corresponds to decreasing χ_PC_ and as the result increasing Δχ. According to eq 11, this will drive the system from Regime I toward the monotonic regime, leading to a reduced extent of reentrant conformation transition by the polymer (Figure 2(a)), exactly as observed in ref (33). The “entropic” model in ref (33) features a size difference between the monomer and solvent as the cosolvent size is varied in-between. This microscopic setting corresponds to having χ_SC_ > 0 and Δχ > 0 (i.e., χ_PS_ > χ_PC_ because of a smaller size difference between monomer and cosolvent). Increasing monomer–solvent size difference will increase both χ_SC_ and Δχ and eventually bring the system inside Regime II according to eq 11. In the meanwhile, reducing cosolvent size toward the size of solvent corresponds to reducing both χ_SC_ and Δχ toward zero. According to eq 11, this acts to drive the system from Regime II toward the monotonic regime and reduce the extent of reentrant transition, as observed in ref (33). Notably, in Regime II, the onset of preferential adsorption does not commence the reentrant conformation transition, which can be seen in Figures 2(d) and 3(d) for the case (Δχ = 3, χ_SC_ = −1). At last, in the model that features strong solvent–solvent energetic attractions, this setting corresponds to having χ_SC_ > 0 and Δχ > 0. Increasing solvent–solvent attraction strength results in greater χ_SC_ and Δχ, which will eventually bring the system inside Regime II. Keeping everything else unchanged, increasing solvent-cosolvent energetic attraction strength will act to reduce χ_*SC*_ and as the result χ_SC_ + Δχ. According to eq 11, this will drive the system toward the monotonic regime and reduce the extent of reentrant transition, again in agreement with ref (33). The above comparisons demonstrate χ_SC_ + Δχ acting as the underlying universal parameter in models with differing microscopic details in regulating polymer cononsolvency.

Although this study is based on the Gaussian chain model, we expect that the qualitative conclusions still apply when considering real polymers with finite chain length and excluded volume effects. This is based on two considerations: (1) As long as the polymer chain length exceeds several persistence lengths, the bending rigidity of real polymers can be renormalized into an effective Kuhn length in the Gaussian chain model; and (2) polymer cononsolvency concerns mostly with the long-length-scale properties of polymers, i.e., mean-square end-to-end distance as the function of solution composition. At this length scale, the averaged effect of microscopic interactions between monomers, responsible for the excluded volume effects in real polymers, can be modeled using effective pseudo potentials such as the one shown in eq 8. Furthermore, calculations in this study are conducted at the zeroth order with the intention of showcasing the viability of formulating general cononsolvency conditions using generic considerations. Improvements can be systematically conceived toward more accurate descriptions by incorporating higher-order effects into the calculations. One possible improvement is to still assume concentration-independent χ but retain the term χ_PSC_ϕ_P_ϕ_S_ϕ_C_ in eq 2. By adsorbing χ_PSC_ϕ_P_ϕ_S_ϕ_C_ into χ_PC_ϕ_P_ϕ_C_, the effective parameter χ_PC_^eff^ ≡ χ_PC_ + χ_PSC_ϕ_S_ contains the first-order correction to χ_PC_ due to the solvent-mediated effects. This may help address the atypical high threshold values in Δχ obtained by the current calculations. A full treatment of concentration-dependent χ will have to rely on the microscopic details for determining the exact form of the dependence. In its current form, the generic model can be readily applied to studies of cosolvent effects in more complex systems, such as the solution-phase polymer self-assemblies, which are of practical importance but difficult to address directly using the chemistry-specific atomistic models. Studies along these lines will be reported in future publications.

## 5 Appendix

### 5.1 Expansion of Δ*f*^ex^ and χ-Parameters

Assuming no volume change upon mixing (i.e., *V* = ∑_*i*_*V*_*i*_^(0)^ with *V*_*i*_^(0)^ being the volume of the *i*-component in its pure state), the free energy of mixing per unit volume is given by
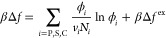
13where *v*_*i*_ is the volume of an *i*-type monomer, ϕ_*i*_ = *V*_*i*_^(0)^/*V* is the volume fraction of the *i*-type monomer in the mixture , and βΔ*f*^ex^ is the excess free energy of mixing given as
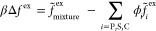
14with *f̃*_*i*_^ex^ being the excess free energy per unit volume of the pure-*i* state. As a mixing property, *β*Δ*f*^ex^ must vanish when ϕ_*i*_ = 1 and reduce to the excess free energy of mixing of a binary mixture when ϕ_*i*_ = 0 (*i* = P or S or C). For that, one can argue that *β*Δ*f*^ex^ can be expanded into series of binary ϕ_*i*_^*m*^ϕ_*j*_^*n*^ and ternary ϕ_*i*_^*m*^ϕ_*j*_^*n*^ϕ_*k*_^*p*^ terms, with *m*, *n*, *p* ≥ 1 being integers.^34^ The high-order terms in the expansion can be adsorbed into the leading-order terms by introducing ϕ-dependent coefficients χ_*ij*_ and χ_*ijk*_, which leads to the concise expression for *β*Δ*f*^ex^ shown in eq 2.

The nature of the χ-parameters in eq 2 can be inferred by a similar expansion of *f̃*_mixture_^ex^. Recognizing that *f̃*_mixture_^ex^ must recover the excess free energy of the pure-*i* state when ϕ_*i*_ = 1 (*i* = P or S or C), *f̃*_mixture_^ex^ can be expanded into the following form^34^
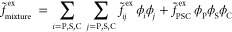
15

Considering that , the double sum can be interpreted as the additive effects of mixing, with the expansion coefficients *f̃*_*ij*_^ex^ (*i* ≠ *j*) and *f̃*_*ii*_^ex^ = *f̃*_*i*_^ex^ carrying the meaning of per unit volume excess free energy due to *i*–*j* cross interactions and *i*–*i* interactions, respectively. The *f̃*_PSC_^ex^ term corrects for the “non-additive” effects such as the free volume and contact surface dissimilarities,^35^ or the formation of coordinated polymer–solvent-cosolvent complexes.^36^ By substituting eq 15 into eq 14 and comparing with eq 2, one obtains χ_*ij*_ = 2*f̃*_*ij*_^ex^ – *f̃*_*i*_^ex^ – *f̃*_*j*_^ex^ and χ_*ijk*_ = *f̃*_*ijk*_^ex^ from which their free-energy nature becomes evident. More specifically, the χ-parameters encode all (both enthalpic and entropic) effects of mixing due to, e.g., dispersion interactions, dissimilarities in the excluded volume, and chain conformation changes, etc., and are generally composition dependent.

###  from the Flory–Huggins Theory

5.2

Consider a binary solution of solvent (S) and cosolvent (C) in osmotic equilibrium with a ternary solution consisting of S, C, and polymer (P), the osmotic pressure is then defined through equating the chemical potentials of the diffusible components in the two systems, i.e.
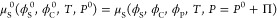
16
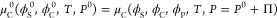
17with , , , and , and *G*^0^ and *G* being the Gibbs free energy of the binary and ternary systems, respectively. Assuming no volume change upon mixing and segmental volume *v*_0_ = 1, *G*^0^ and *G* can be written as
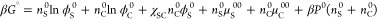
18
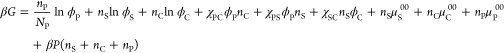
19respectively, where  and μ_*i*_^00^ is the chemical potential of pure component *i*. Combining eqs 16–19, the chemical potentials of the diffusible components and the equations for osmotic pressure are obtained as
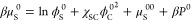
20
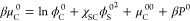
21
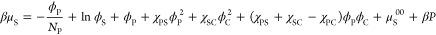
22
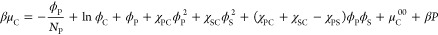
23
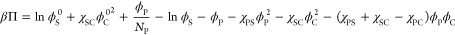
24
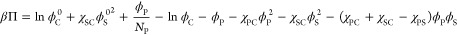
25

In principle, Π can be solved in terms of ϕ_S_, ϕ_C_, and ϕ_P_ from eqs 24 and 25 together with ϕ_S_^0^ + ϕ_C_^0^ = 1. To the best of our efforts, solutions are obtainable only via density expansions. For ϕ_P_ → 0, substitute (37) into eqs 24 and 25 and apply Taylor series for ln(*x*); the expansion coefficients *A*_1_, *A*_2_ are determined (by matching the corresponding coefficients of the two resulting polynomials) as
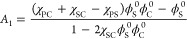
26
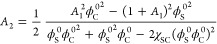
27Following this, the osmotic second virial coefficient  can then be identified as
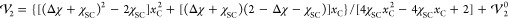
28where , Δχ ≡ χ_PS_ – χ_PC_, and .

It is noteworthy that using a similar approach in ref (8) obtains an expression for  that differs from eq 28. Here, we explain the cause of this disparity. Following the definitions in eqs 16 and 17, but express μ_S_^0^ and μ_S_ (similarly for μ_C_^0^ and μ_C_) using the Helmholtz free energy, i.e.,  and , where Δ*F*^0^ and Δ*F* are the Helmholtz free energy of mixing of S + C and P + S + C mixtures, respectively. Plugging μ_S_^0^ and μ_S_ into eq 16, one obtains
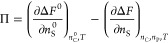
29

Changing variables from Δ*F*(*n*_S_, *n*_C_, *n*_P_, *T*) to Δ*F*(*V*, ϕ_P_, *x*_C_, *T*) by applying chain rule to the second term, i.e., , and substituting into eq 29, one obtains
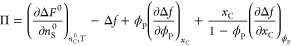
30where . Substitute the Taylor expansion  into the second and third term in eq 30, and one obtains
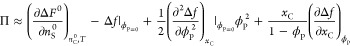
31

The first two terms in eq 31 do not contribute to . In the special case of a binary P + S solution (i.e., *x*_C_ = 0), eq 31 yields  with  being the excluded volume parameter. This corresponds to the result reported in ref (8). However, for ternary solutions, the last term in eq 31 does not vanish and will also contribute to , which was neglected in ref (8). This results in conclusions that are different from those in the current study.

### 5.3 Polymer End-to-End Distance and Radial Distributions from the Self-Consistent Field Calculations

The extended Percus test-particle method^38^ is applied in the SCF calculations to obtain the polymer end-to-end distance and the radial distributions. We consider a system of volume *V* consisting of a single polymer (P) chain tethered to the origin by the *t*-th segment mixed with solvent (S) and cosolvent (C). The partition function of the system is given by
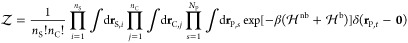
32with  and  being given in eqs 7 and 9. By labeling the chain section 1 ≤ *s* < *t* as “L” and the section *t* < *s* ≤ *N*_P_ as “R”, and defining ρ̂_L,c_ (**r**) ≡ ∑_*s*=1_^*t*–1^ δ (**r** – **r**_P,*s*_), ρ̂_R,c_ (**r**) ≡ ∑_*s*=*t*+1_^*N*_P_^ δ (**r** – **r**_P,*s*_), after integrating out the δ-function, the partition function of the system becomes
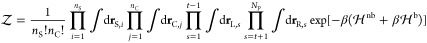
33with
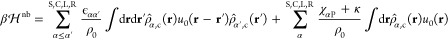
34
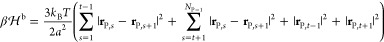
35where ϵ_α_α′ = χ_α_α′ + κ for α ≠ α′ and κ for α = α′. Following the standard Hubburd-Stratonovich transformation, the SCF equations are obtained under saddle-point approximation as
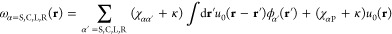
36
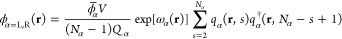
37
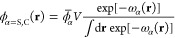
38where *q*_α=L,R_(**r**, *s*) and *q*_α=L,R_^†^(**r**, *s*) are the forward and backward propagators for the L and R chain sections that satisfy the recursive formula
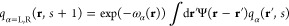
39a
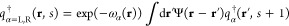
39bwhere 1 ≤ *s* ≤ (*t* – 1) for α = L and *t* ≤ *s* ≤ *N*_P_ – 1 for α = R, and  is the bond transition factor. The initial conditions for eqs 39a and 39b are given by *q*_L_(**r**, 1) = exp(−ω_L_(**r**)), *q*_R_(**r**, *t*) = δ(**r** – **0**) and *q*_L_^†^(**r**, *t*) = δ(**r** – **0**), *q*_R_^†^(**r**, *N*_P_) = exp(−ω_R_(**r**)), respectively. The partition functions of the L and R sections can then be calculated as *Q*_L_ = ∫d**r***q*_L_(**r**, 1)*q*_L_^†^(**r**, 1) and *Q*_R_ = ∫ d**r***q*_R_(**r**, *N*_P_)*q*_R_^†^(**r**, *N*_P_).

The SCF equations are solved in a spherical coordinate. We assume that the fields ω_α_(*r*, ψ, θ) and ϕ_α_(*r*, ψ, θ) as well as the chain propagators *q*_α_(*r*, ψ, θ, *s*) and *q*_α_^†^ (*r*, ψ, θ, *s*) are ψ- and θ-independent. The integration in the equation for ω_α_(**r**) can be re-expressed as
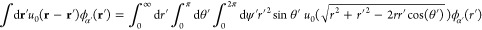
40where we have set ψ = 0 and θ = 0 without the loss of generality. Substituting into eq 40 the expression for *u*_0_(**r** – **r**′) given by eq 8 and carrying out the integrations over ψ and θ, after some reorganization we obtain
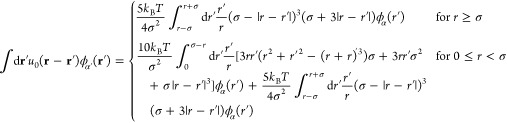
41Following a similar procedure, eq 39a can be solved as
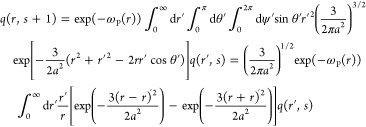
42In calculations, a cutoff radius L_r_ is used to carry out the integration, i.e., ∫_0_^∞^ ≈ ∫_0_^L_r_^, with L_r_ being large enough such that the solution composition at *r* = L_r_ approaches that of a binary S + C solvent mixture. A similar expression can also be derived for *q*^†^(*r*, *s*) starting from eq 39b.

To calculate the end-to-end distance of polymer chain , we set *t* = 1. After solving the SCF equations, the end-to-end distance can be calculated as
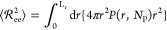
43where *P*(*r*, *N*_P_) is the probability of the free end-segment of the polymer chain being located at a radial distance *r* from the origin (where the first segment of polymer chain is tethered), given by
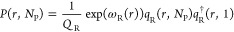
44The radial distributions of the α-type segments about a polymer segment is calculated as
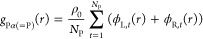
45
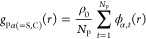
46where ϕ_α,*t*_(*r*) denotes ϕ_α_(*r*) that is calculated with the *t*-th segment being the tethering segment.

### 5.4 Details of Monte Carlo Simulations

Monte Carlo simulations in this study are performed under the canonical (NVT) ensemble. A polymer chain consisting of 80 connected spherical segments of diameter σ = 1 is placed inside a cubic simulation box of dimension *L*_*x*_ = *L*_*y*_ = *L*_*z*_ = 20, together with *n*_S_ solvent and *n*_C_ cosolvent segments. The total number of solvent and cosolvent segments *n*_S_ + *n*_C_ = 63823 is fixed throughout all simulations, whereas the respective values of *n*_S_ and *n*_C_ are adjusted to reflect the solvent mixture composition. Setting *k*_B_T = 1, the nonbonded interaction potential between segment *i* and *j* located at **r**_*i*_ and **r**_*j*_ is calculated as
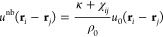
47with κ = 16, χ_*ij*_ = 0 for *i* = *j*, and *u*_0_(**r** – **r**′) taking the form shown in eq 8. In addition, a bonding potential
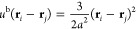
48is applied if the two segments are connected on a polymer chain. Conventional hopping trial moves are performed to equilibrate and sample the polymer configurations. In each trial move, a segment *i* located at **r**_*i*_ = **r**_*i*_^o^ is randomly picked for attempting a trial displacement of Δ**r** = Δ*r*_*x*_**x** + Δ*r*_*y*_**y** + Δ*r*_*z*_**z**, with Δ*r*_α_ (α = *x*, *y*, *z*) uniformly distributed in (−σ/2, σ/2). The change in Hamiltonian due to this displacement is calculated as
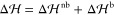
49where  and . The trial move is accepted or rejected according to the Metropolis acceptance criterion
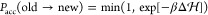
50
